# Subacute Exposure to Low Pb Doses Promotes Oxidative Stress in the Kidneys and Copper Disturbances in the Liver of Male Rats

**DOI:** 10.3390/toxics11030256

**Published:** 2023-03-09

**Authors:** Dragana Vukelić, Aleksandra Buha Djordjevic, Milena Anđelković, Evica Antonijević Miljaković, Katarina Baralić, Katarina Živančević, Petar Bulat, Jelena Radovanović, Danijela Đukić-Ćosić, Biljana Antonijević, Zorica Bulat

**Affiliations:** 1 Department of Toxicology “Akademik Danilo Soldatović”, Faculty of Pharmacy, University of Belgrade, 11221 Belgrade, Serbia; 2 Health Center Kosovska Mitrovica, 38220 Kosovska Mitrovica, Serbia; 3 Institute of Physiology and Biochemistry “Ivan Djaja”, Center for Laser Microscopy, Faculty of Biology, University of Belgrade, Studentski trg 16, 11158 Belgrade, Serbia; 4 Faculty of Medicine, University of Belgrade, 11000 Belgrade, Serbia; 5 Serbian Institute of Occupational Health, 11000 Belgrade, Serbia; 6 Department of Radiobiology and Molecular Genetics, “Vinča” Institute of Nuclear Sciences-National Institute of the Republic of Serbia, University of Belgrade, 11000 Belgrade, Serbia

**Keywords:** oxidative stress, essential elements, Pb exposure, benchmark modelling, dose–response

## Abstract

Recent data indicate that lead (Pb) can induce adverse effects even at low exposure levels. Moreover, the corresponding mechanisms of low Pb toxicity have not been well identified. In the liver and the kidneys, Pb was found to induce various toxic mechanisms leading to organ physiological disruption. Therefore, the purpose of the study was to simulate low-dose Pb exposure in an animal model with the aim of assessing oxidative status and essential element levels as the main mechanism of Pb toxicity in the liver and kidneys. Furthermore, dose–response modelling was performed in order to determine the benchmark dose (BMD). Forty-two male Wistar rats were divided into seven groups: one control group, and six groups treated for 28 days with 0.1, 0.5, 1, 3, 7, and 15 mg Pb/kg b.w./day, respectively. Oxidative status parameters (superoxide dismutase activity (SOD), superoxide anion radical (O_2_^−^), malondialdehyde (MDA), total sulfhydryl groups (SHG), and advanced oxidation protein products (AOPP)) and Pb, copper (Cu), zinc (Zn), manganese (Mn), and iron (Fe) levels were measured. Lowering Cu levels (BMD: 2.7 ng/kg b.w./day), raising AOPP levels (BMD: 0.25 µg/kg b.w./day) in the liver, and inhibiting SOD (BMD: 1.3 ng/kg b.w./day) in the kidneys appear to be the main mechanisms of Pb toxicity. The lowest BMD was derived for a decrease in Cu levels in liver, indicating that this effect is the most sensitive.

## 1. Introduction

Lead (Pb) is one of the most persistent hazardous contaminants in the environment, posing a serious public health risk. It is a multi-organ toxin that affects almost all organs, including the brain, kidneys, liver, and reproductive organs [1,2,3]. Lead exposure primarily occurs through ingesting tainted food or water, or inhaling Pb-contaminated air [4,5]. After being absorbed, Pb is conjugated with glutathione in the liver and distributed between blood and tissues, and a small amount is excreted by the kidneys, so Pb builds up in various body tissues and harms a variety of macromolecules and organelles [6,7]. Epidemiological studies conducted on workers exposed to Pb indicate a connection between Pb exposure and the induction of certain liver enzymes, increased plasma cholesterol levels, disrupted glucose homeostasis, and thickening of the gallbladder wall [8,9]. Chronic exposure to high doses of Pb could cause permanent alterations in the kidneys, including interstitial fibrosis, tubular atrophy, glomerular sclerosis, and eventually renal failure [10,11]. On the other hand, the signs of chronic low-dose Pb poisoning in humans are usually modest, and many individuals remain asymptomatic. Furthermore, chronic low-dose Pb exposure in humans has also been linked to gout and hypertension development trough renal and nonrenal mechanisms [12]. 

In the liver and the kidneys, Pb was found to induce oxidative stress by inducing reactive oxygen species, leading to oxidative damage of crucial molecules, proteins, nucleic acids, and lipids [13]. Although Pb is not a Fenton’s metal, it can induce oxidative damage, indirectly elevating the level of free Fe which acts as a Fenton’s metal. As a divalent cation, its chemical configuration allows Pb to mimic essential cations in physiological processes, such as calcium (Ca^2+^), zinc (Zn^2+^), copper (Cu^2+^) and manganese (Mn^2+^) [14,15]. Most of them are essential for proper enzyme activity as cofactors or for regular cell membrane signal transduction [16]. The elevation of Fe in the blood occurs due to Pb displacement from the hemoglobin molecule (plumbenia). By mimicking and displacing essential cations which are important cofactors of antioxidant enzymes, Pb decreases their activity by increasing oxidative stress in the tissues. However, hepatotoxic and nephrotoxic mechanisms in the case of low, environmentally relevant doses of Pb are still not clear [8,17].

Lead’s toxic potential has been extensively studied for many years. During widespread use of Pb in industry, especially after 1970s, humans were exposed to high lead levels that were reflected in blood Pb levels (BLLs) of 100 µg/dL, or even higher [18,19]. Nowadays, after the ban of Pb use in the many products, the BLL in the non-occupationally exposed general population is usually lower than 5 µg/dL (set as reference level by the Center for Disease Control and Prevention) [20,21]. In order to derive the point of departure for hazardous chemicals, for regulatory purposes, a novel benchmark approach has been developed. It is an advanced method that uses software modelling for toxic dose–response analysis [22,23].

The purpose of the present study was to simulate a low-dose subacute Pb exposure scenario in animal model, with the aim of obtaining relevant BLLs for environmental exposure. After determination of hepatotoxic and nephrotoxic effects, the aim was to determine the benchmark dose for such effects, which might be useful in further human health risk assessment and safety evaluation of low-dose Pb exposure.

## 2. Materials and Methods

### 2.1. Chemicals

All chemicals used for oxidative status analyses and metal analyses were p.a. quality, and were purchased from Sigma Aldrich, Germany or Scientific Fisher, Germany. Lead (II) acetate trihydrate (Pb (CH_3_CO_2_)_2_ • 3H_2_O), Alkaloid Skopje Macedonia, was used for making solutions for experimental animal treatment.

### 2.2. Animals and Experimental Study Design

The study was conducted on forty-two male albino Wistar rats (six weeks old) purchased from Military Medical Academy, Belgrade. The rats were randomized in seven groups (n = 6) and acclimatized for one week in the animal room at the Faculty of Pharmacy, University of Belgrade, under relative humidity of 40–60%, a temperature of 25 ± 3 °C, and a 12 h light–dark cycle. After acclimatization, six groups were treated with rising doses of Pb (0.1, 0.5, 1, 3, 7, 15 mg Pb/kg body weight (b.w.)/day), while the control group received distilled water only. Oral gavage was performed every morning for a period of 28 days. The doses were chosen to mimic environmentally realistic and low-subacute exposure to Pb with the aim of obtaining BLLs that have been reported in Pb-exposed human populations [18,24,25,26]. Twenty-four hours after the last dose, the rats were humanely sacrificed. The liver and the kidneys were taken, extensively washed (ice cold 0.9% NaCl) and dry-weighed. The relative organ weight (%) was calculated as organ weight (g)/final body weight (g) × 100. The samples of organs were dissected and appropriately stored for oxidative status (−80 °C), and metal (−20 °C). The study was approved by the Ethical Committee on Animal Experimentation of the University of Belgrade Faculty of Pharmacy (No. 323-07-11822/2018-05), and was carried out in accordance with the United Kingdom Animal (Scientific Procedures) Act 1986 and the EU Directive 2010/63/EU for animal experiments.

### 2.3. Metal Analyses

The preparation of samples for metal analyses was carried out using a microwave-assisted digestion system (Milestone, Start D SK-10T, Milestone Srl, Sorisole, Italy). Weighted samples were digested using 7 mL concentrated HNO_3_ (69%) and 1 mL concentrated H_2_O_2_ (30%) under conditions suggested by the manufacturer. After digestion, the cooled samples were filled up to 25 mL with deionized water. The determination of Pb was performed using a graphite furnace atomic absorption method, while essential metals were determined using a flame atomic absorption method on an AAS GTA 120 graphite tube atomizer, 200 series AA, Agilent Technologies, Santa Clara, CA, USA. The accuracy of the analyses was checked using standard reference materials (SRM) whole blood Level 2 (SeronormTM, Sero, Billingstad, Norway) and 1577c—Bovine liver (LGS Standard, London, UK). 

### 2.4. Oxidative Status Analyses

Preparation of tissue samples for oxidative status analyses consisted of homogenization with cold 0.1 mol/L phosphate buffer (pH 7.4) in a 1:9 weight-to-volume ratio, using a T10 basic Ultra-Turrax homogenizer (IKA, Staufen, Germany). With the aim of obtaining post-mitochondrial supernatant, the homogenates were centrifuged at 800× *g* for 10 min and then at 9500× *g* for 20 min (+4 °C). All oxidative status parameters were determined in the post-mitochondrial supernatant. The rate of superoxide anion radical (O_2_^−^) formation was determined using the method described by Auclair and Voisin [27]. The Misra and Fridovich method [28] was used for superoxide dismutase activity (SOD) determination. The total oxidative status (TOS) was measured based on the Erel-optimized spectrophotometric method [29]. The malondialdehyde (MDA) concentration was determined using a spectrophotometric method with thiobarbituric acid, and the results are expressed as μmol/g protein [30]. Total sulfhydryl groups (SHG) were calculated using the spectrophotometric method with 5,5′-dithiobis-2-nitrobenzoic acid (DTNB), as described by Ellman [31]. The method published by Witko et al. [32] was used for determination of advanced oxidation protein products (AOPP). All parameters are expressed on protein levels that were measured using the Bradford method [33].

### 2.5. Benchmark Dose Modeling

PROAST 70.1 software (https://www.rivm.nl/en/proast, accessed on 22 August 2020) was used to conduct benchmark dose–response modeling (Dutch National Institute for Public 132 Health and the Environment, RIVM). For continuous data, a benchmark response (BMR) of 5% was utilized, as recommended by the Scientific Committee of the European Food Safety Authority (EFSA) at a 90% confidence level [23]. External dosages, BLL and Pb levels in liver and kidneys tissues were all examined as doses for all parameters obtained in the research. The benchmark dose interval (BMDI) was determined using the program, which included lower (BMDL) and upper (BMDU) BMD. The model-averaging method in PROAST software combines all available models into one, which is then applied in data processing [34].

### 2.6. Statistical Analyses

GraphPad Prism8 software (GraphPad Software Inc., San Diego, CA, USA) was used for statistical analyses and graph-making. If data passed a normality and homogeneity of variance check, the data were presented as mean and standard deviation (S.D.), and analyzed using a one-way ANOVA followed by Fisher’s LSD test. If not, the data were presented as median and ranges (minimum-maximum), and the Kruskal-Wallis test followed by the Mann–Whitney U test was used. The level of statistical significance was set at *p* < 0.05. 

## 3. Results

### 3.1. Relative Organ Weight

Relative liver and kidney weights are presented in Table 1. The results show that there are no statistically significant differences in the organ weight of rats between groups.

### 3.2. Lead and Essential Metal Status in Rats’ Liver and Kidneys

**Figure 1:**
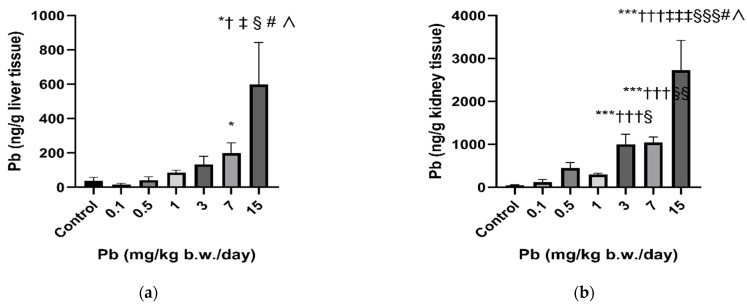
Liver (**a**) and kidney (**b**) Pb levels in Wistar rats subacutely exposed to low levels of Pb over a period of 28 days. Results are presented as means ± SD. * ^† ‡ § # ∧^ *p* < 0.05; ^§§^ *p* < 0.01; *** ^††† ‡‡‡ §§§^ *p* < 0.001 compared to control, 0.1, 0.5, 1, 3, and 7 group, respectively, using ANOVA followed by Fisher’s LSD test.

Lead tends to accumulate in the rat liver and kidney (Figure 1). In the liver, statistically increased Pb levels were determined in the groups treated with 7 and 15 mg Pb/kg b.w./day compared to controls, and in the case of the highest Pb dose of 15 mg Pb/kg b.w./day, in all treated groups. In the case of the kidneys, a statistically significant increase in Pb levels compared to control was detected in groups treated with 3, 7, 15 mg Pb/kg b.w./day. In both organs, a dose-dependent increase in the bioaccumulation of Pb was observed.

A trend of decreasing Cu levels in the liver, with the lowest value in the highest dose group, can be observed (Table 2). In the liver, no statistically significant difference was observed in values of other essential elements (Zn, Fe, Mn) in relation to the control value. In the kidneys, there were no statistically significant changes in essential elements compared to the control group.

### 3.3. Oxidative Status in Rats’ Liver and Kidney Tissues

In the liver, an increasing trend in AOPP levels was observed in the treated groups of rats (Table 3). In the treated groups, induction of SOD enzyme activity was also observed, with the highest value in the group treated with the highest dose of 15 mg Pb/kg b.w./day. The level of MDA varied irregularly between groups. In all treated groups, inhibition of the renal enzyme’s SOD activity occurred (Table 3). A slight decrease in MDA levels was observed in some treated groups. Other oxidative stress parameters remained unchanged from the control. 

### 3.4. Benchmark Dose Modelling

**Figure 2:**
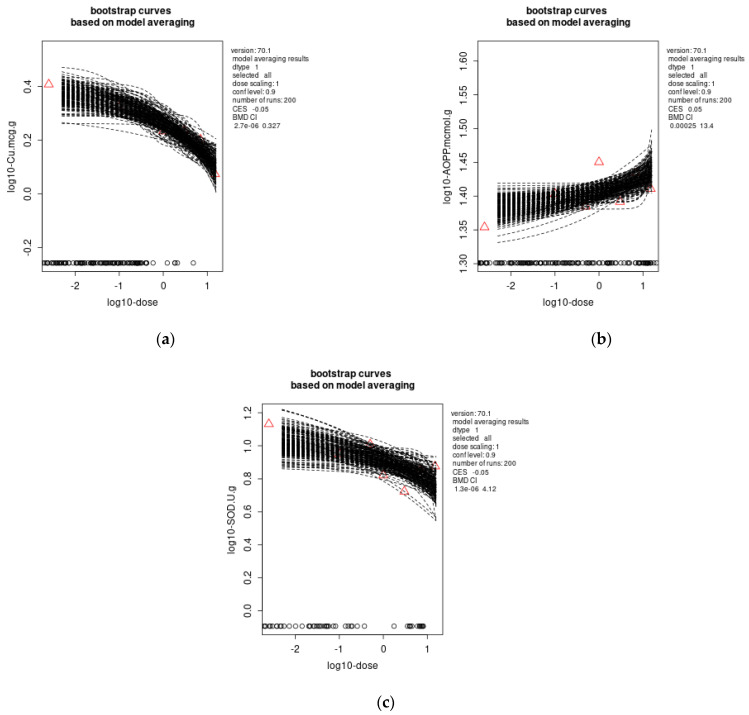
Benchmark dose modeling of liver Cu (**a**) and AOPP (**b**) levels, and kidneys’ SOD (**c**) activity as a response to the external Pb dose (mg/kg b.w./day), using a model averaging method with 200 iterations, PROASTweb70.1 software (https://proastweb.rivm.nl/). The red triangles represent the medians.

**Figure 3:**
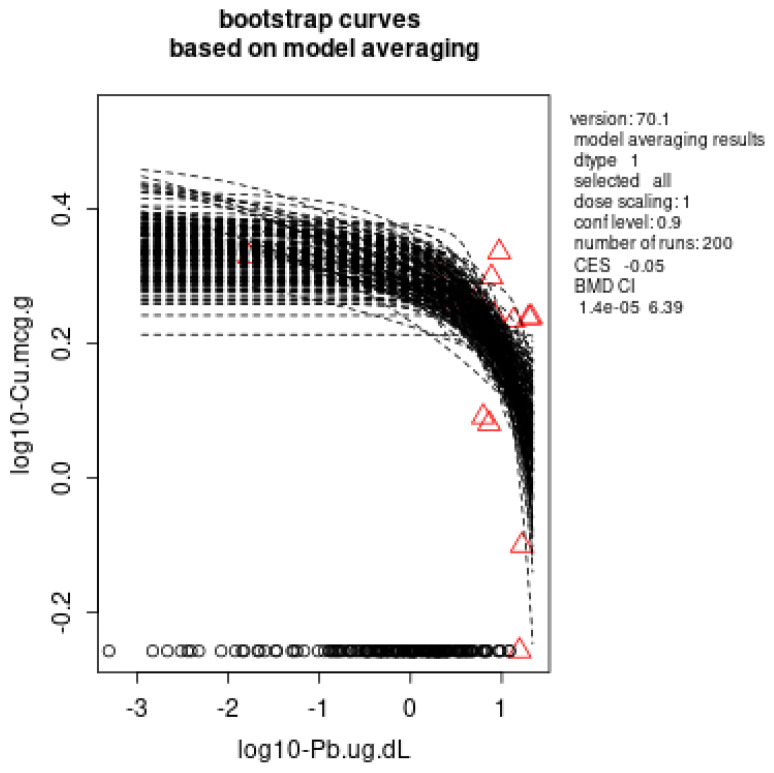
Benchmark dose modeling of liver Cu levels as a response to the internal Pb dose–BLL (µg/dL) using a model averaging method with 200 iterations, PROASTweb70.1 software (https://proastweb.rivm.nl/). The red triangles represent the medians.

**Figure 4:**
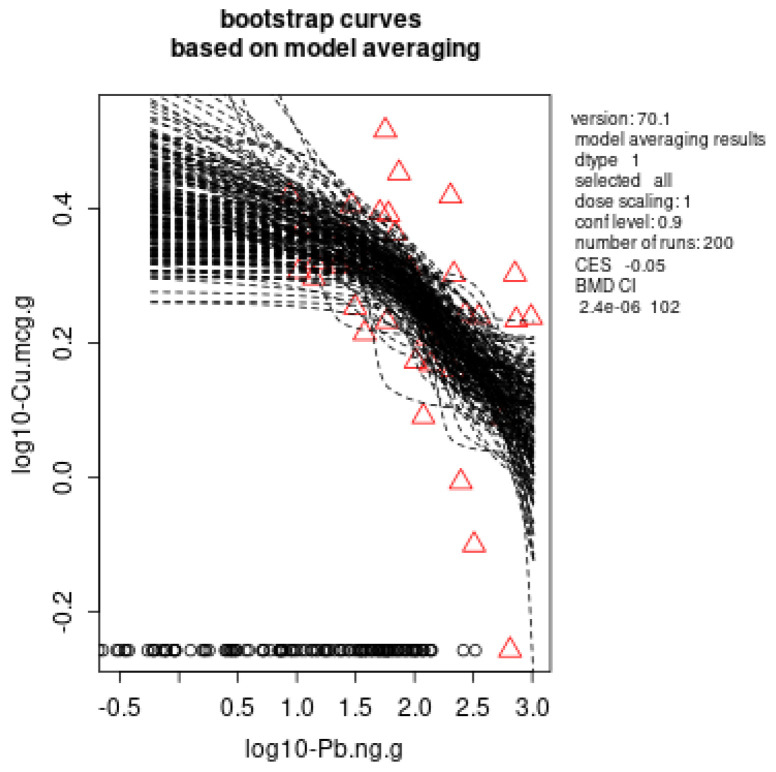
Benchmark dose modeling of the liver Cu levels as a response to the internal Pb dose–liver tissue level (µg/g) using a model averaging method with 200 iterations, PROASTweb70.1 software (https://proastweb.rivm.nl/). The red triangles represent the medians.

In the liver, dose dependence for the Pb effects was observed for Cu with external dose–response, BMDL: 2.7 × 10^−6^ mg Pb/kg b.w./day (Figure 2a) and AOPP with external dose–response, BMDL: 0.00025 mg Pb/kg b.w./day (Figure 2b). In case of internal dose (BLL)-response, BMDL: 1.4 × 10^−5^ µg/dL (Figure 3) and internal dose (ng Pb/g liver)-response, BMDL: 2.4 × 10^−6^ ng Pb/g (Figure 4) was determined for the Cu decrease. 

The external dose–response relationship for the effects of Pb in the kidneys was detected for SOD activity (Figure 2C), with BMDL: 1.3× 10^−6^ mg Pb/kg b.w./day. 

For all other investigated parameters in the liver and the kidneys, the dose dependence was not obtained.

## 4. Discussion

In the present study, we have examined the hepatotoxic and nephrotoxic effects of low Pb doses, reaching a BLL similar to general exposed population [35,36]. The six dose groups allowed ideal statistical conditions for benchmark dose analysis. Our data suggested that the Pb treatment did not affect liver and kidney weight. Lowering Cu levels in the liver (BMDL: 2.7 × 10^−6^ mg Pb/kg b.w./day), along with higher AOPP liver levels (BMDL: 0.00025 mg Pb/kg b.w./day), and inhibition of SOD (BMDL: 1.3 × 10^−6^ mg Pb/kg b.w./day) in the kidneys might be the most sensitive mechanisms of low-dose Pb toxicity in these organs.

Lead exposure has been found to affect all organs, including the blood, liver, kidneys, heart and brain, affecting their physiological structure and function [2,35,37,38,39]. Effects on the liver and kidneys are key points in the evaluation of the safety of existing and new substances. The main parameters in the serum that are usually used for the evaluation of liver function are liver enzymes’ activity and lipid profile parameters [40]. In our previously published study, we have reported that low lead exposure led to dysregulation of lipid profile in subacutely exposed rats, while liver enzymes remained in normal ranges [35]. Both Cu excess and deficiency in the liver can induce clinical problems, since it is a component of various enzymes that are required for health and wellbeing. Liver Cu levels homeostasis are highly regulated, and normally neither a decrease nor an excess buildup of Cu occurs due to effective physiological regulation processes [41,42]. The liver regulates the elimination of acquired Cu from the body through bile. Along with Zn, Cu has high affinity for SHG of metallothionines. Furthermore, as a cofactor, Cu is crucial for the function of several enzymes: SOD, mitochondrial monoamine oxidase, tryptophon-2,3-dioxygenase, cytochrome c oxidase, ceruloplasmin, and hephaestin [41,42]. Lead has been shown to induce Cu deficiency in animal studies [43] and humans [44], while Cu supplementation in animal studies has been proven to have protective effects on Pb toxicity [45]. In the present study, low Pb doses induced an external and internal (BLLs and tissue Pb levels) dose-dependent decrease in Cu in the liver. The derived BMDs were: 2.7× 10^−6^ mg/kg b.w./day for an external dose, 1.4 × 10^−5^ µg/dL for an internal dose (BLL), and 2.4 × 10^−6^ ng/g Pb for an internal dose (ng Pb/g liver tissue). The decrease in Cu may probably affect all previously mentioned biological processes in the liver, possibly leading to disruption of liver function. A potential mechanism by which Pb decreases Cu could be competition between Pb and Cu for several biological process, including transport through membranes or competition for active seats in biologically active proteins. By altering ion homeostasis, Pb causes many of its harmful effects. This disruption happens when Pb replaces other metal ions such as iron, calcium, zinc, magnesium, selenium, and manganese [14,16].

Another important mechanism of Pb toxicity is the induction of oxidative damage in various tissues [13]. Our results have shown induction of SOD enzyme activity and dose-dependent increases in AOPP levels in the liver, with a derived BMDL of 0.00025 mg Pb/kg b.w./day indicating oxidative stress induction in the liver. Advanced oxidation protein products are dityrosine-containing cross-linked protein products that have been shown to be good indicators of protein oxidation. In 1996, AOPP were first identified in the plasma of chronic uremic patients as new oxidative stress indicators [32]. AOPP are transported by oxidized plasma proteins and bind to the high-density lipoprotein (HDL) scavenger receptor class B type I; thus, they are classified as HDL receptor antagonists [46]. The induction of liver SOD enzyme activity in the present study might be a protective mechanism on oxidative stress induction, keeping in mind that production of free radicals can stimulate antioxidant enzymes’ activity or inhibit it [47]. Similar to our results, Barregard et al. have shown (40 days, 10.39 mg Pb/kg b.w./day) an increase in SOD activity in the liver and a decrease in SOD activity in the kidneys of rats [11]. In studies wherein higher doses of Pb were tested, inhibition of SOD, catalase (CAT) and glutathione peroxidase (GPx) have been documented. For example, in the 5-day long study on intraperitoneal application of Pb-acetate to Wistar rats, a dose of 20 mg/kg b.w./day induced a decrease in the liver’s SOD activity [48]. Another animal study on Wistar rats (100 mg/kg b.w./day, per os, 60 days) also resulted in a decrease in SOD activity, CAT activity and glutathione levels, while MDA increased in the liver, compared to the untreated group [49]. 

Lead excretion from organisms and lead’s ability to be distributed and accumulated in the kidneys allows for direct toxic effects. Pb has been shown to induce kidney damage through several mechanisms, including general oxidative stress induction, essential cation interaction, inflammation, and induction of glomerular and tubular cell apoptosis. Some of its additional mechanisms are changes in renal gangliosides (plasma membrane lipids that play a role in the control of glomerular filtration), changes in renal vascular tone, and alterations in the renin–angiotensin–aldosterone hormonal system [8]. In our previous study, we showed that low subacute lead exposure did not significantly impact the serum nephrotoxicity markers of creatinine and urea levels [35]. A recent study by Kwon et al. suggested a new mechanism following Pb exposure erythrophagocytosis in renal tubular cells, which might greatly increase nephrotoxicity [17]. The observed data in our study have shown a dose-dependent decrease in renal SOD activity in Pb-treated animals, with derived BMDL: 1.3 × 10^−6^ mg Pb/kg b.w./day. SOD activity has been disrupted probably due to direct inhibition of SOD by Pb, or due to the lower capacity of SOD synthesis. The SOD enzyme plays a key role in oxidative stress resistance, and it is responsible for control of potential over O_2_^−^ production in the cell. It catalyzes the reaction of O_2_^−^ dismutation into H_2_O_2_ and oxygen [50]. Similar to our results, in the study on male Wistar rats, Pb-acetate (20 mg/kg, i.p.) induced a decrease in renal antioxidant enzyme activity (SOD, catalase, and glutathione peroxidase) after seven days of treatment [51], and inhibition of renal SOD was noticed in the study on Wistar rats treated with 22.5 mg/kg b.w Pb-nitrate over a period of 28 days [52]. The other investigated parameters of oxidative status in the kidneys did not change significantly. Moreover, essential elements levels did not change. 

The majority of Pb’s harmful effects are thought to manifest at a BLL of 5 µg/dL or lower. As a result, the Centers for Disease Control and Prevention (CDC) is contemplating decreasing the reference levels for BLL in children from 5 g/dL to 3 g/dL [18]. Our findings support this, keeping in mind that our computed BMDL results were in the range of micrograms Pb per kg b.w./day. In our previous studies, the lowest BMD for Pb for neurotoxic effects was 4.5 × 10^−6^ mg Pb/kg b.w./day for induction of TOS in the brain [2]. This value was also in the range of values obtained in the presented study. Furthermore, for the cardiotoxic effects, the lowest BMD was 2.2 × 10^−6^ mg Pb/kg b.w./day for increases of MDA levels in cardiac tissue, while in case of toxic effects on blood, the lowest BMD was, as in the study in the liver, for a decrease in Cu levels, 1.4 ng/kg b.w./day [35,39]. The obtained results for the decrease in Cu in liver are in correlation with our previous results in blood. Nonetheless, this is to be expected, given that liver is rich in blood and receives over one quarter of the heart’s blood rate, despite making up only few % of the body’s total weight. On the other hand, some epidemiological studies reported the BMD values of Pb for few toxicological endpoints; however, to the best of our knowledge, there are no published values considering hepatotoxic and nephrotoxic endpoints. In the study, performed in China, that included lead-acid battery workers, the authors derived a BMDL of 13.5 µg Pb/dL for a decrease in red blood cells’ concentration, 10.5 µg Pb/dL for a decrease in hemoglobin levels, based on hematological toxicity, and an even tougher threshold of 6.6 µg Pb/dL for micronuclei or 3.5 µg Pb/dL for telomere length, based on genotoxicity [53]. Our research group’s recent publication demonstrated a connection between lead and hormones’ action, indicating a positive association between BLLs and serum insulin levels, with derived BMDs 1.49 and 0.74 µg Pb/dL in males and females, respectively [54]. In our study, the observed internal BMDs (blood lead levels) are lower than the values from epidemiological studies; this strengthens the notion that the blood Pb threshold level might be very low.

## 5. Conclusions

In conclusion, our results strongly suggest the involvement of Cu in low-dose Pb hepatotoxicity. The higher levels of AOPP in the liver also suggest oxidative stress as an important mechanism. In the kidneys, Pb was found to inhibit the SOD antioxidative enzyme activity, so the oxidative stress was found to be the main mechanism in low-dose Pb nephrotoxicity. Benchmark dose modelling showed a dose response for those parameters. The lowest BMD was derived for the decrease in Cu levels in liver, indicating this effect is the most sensitive. Our results might be useful in low-dose Pb exposure risk assessment, and strengthens the notion that the Pb threshold level for negative health effects might be very low. Therefore, exposure to Pb needs to be as low as possible in order to protect human health.

## Figures and Tables

**Table 1:** Relative organ weight of rats subacutely treated with different low Pb doses.

Dose (mg Pb/kg b.w./Day)	Liver (g/100 g Body Weight)	Kidneys (g/100 g Body Weight)
Control	4.13 ± 0.50	0.72 ± 0.06
0.1	4.29 ± 0.32	0.69 ± 0.08
0.5	4.26 ± 0.29	0.66 ± 0.05
1	4.17 ± 0.24	0.66 ± 0.04
3	4.04 ± 0.11	0.64 ± 0.04
7	4.01 ± 0.28	0.69 ± 0.06
15	4.50 ± 0.24	0.72 ± 0.03

The values are presented as means ± standard deviation, N = 6, compared to all other groups, using ANOVA followed by Fisher’s LSD test.

**Table 2:** Essential elements levels in liver and kidneys tissue of Wistar rats subacutely exposed to low levels of Pb.

Liver		Dose (mg Pb/kg b.w./Day)						
		Control	0.1	0.5	1	3	7	15
Cu	Mean	2.58	2.11	1.94 *	1.78 **	1.73 **	1.68 ***	1.29 ***^††‡^
µg/g	SD	0.39	0.19	0.26	0.59	0.26	0.59	0.52
Zn	Mean	25.22	27.58	24.60	26.71	23.28	26.67	22.24
µg/g	SD	2.24	1.63	2.13	5.55	2.56	5.87	4.48
Fe	Mean	88.19	103.5	92.85	92.79	76.57 ^††^	103.1 ^#^	86.89
µg/g	SD	8.12	13.01	17.74	23.04	8.89	13.83	19.49
Mn	Mean	4.21	4.77	2.07 ^†^	2.49	2.15	5.60 ^‡§#^	2.70 ^∧^
µg/g	SD	1.38	1.22	1.19	1.52	1.37	2.42	1.19
Kidney		Control	0.1	0.5	1	3	7	15
Cu	Mean	1.79	1.65	1.88	1.36	2.04	1.38	1.43
µg/g	SD	0.82	0.58	0.52	0.62	0.78	0.49	0.35
Zn	Mean	9.48	8.52	10.27	8.91	11.87	8.02	7.97
µg/g	SD	4.22	2.84	2.92	4.34	3.29	2.51	0.67
Fe	Mean	5.15	6.07	8.93	5.28	8.38	6.14	5.52
µg/g	SD	1.59	2.13	3.99	2.35	2.80	2.25	1.88
Mn	Mean	3.47	1.95	2.41	3.13	2.86	2.40	2.62
µg/g	SD	1.52	0.98	0.33	1.24	1.48	0.58	0.98

* ^† ‡ § # ∧^ *p* < 0.05; ** ^††^ *p* < 0.01; *** *p* < 0.001; compared to control, 0.1, 0.5, 1, 3, 7 group, respectively; ANOVA followed by Fisher’s LSD test.

**Table 3:** Oxidative status in the liver and kidney tissue of Wistar rats subacutely exposed to low levels of Pb.

Liver	Dose (mg Pb/kg b.w./Day)							
		Control	0.1	0.5	1	3	7	15
AOPP	Mean	21.80	24.25	23.10	28.00 ***^‡^	24.60 ^#^	27.05 **^‡^	23.60 ^#∧^
(μmol/min/g)	SD	2.41	2.81	2.85	3.14	0.98	1.94	1.34
O_2_^−^	Mean	32.89	36.47	36.51	34.90	38.07	33.88	39.05
(μmol/min/g)	SD	2.73	3.98	5.91	3.04	1.89	3.75	7.73
SOD	Mean	5.753	10.44 ***	7.29 ^†^	7.30 ^†^	10.31 **^‡§^	7.84 ^†^	13.28 ***^†‡§#∧^
(U/g)	SD	2.63	3.26	2.92	0.94	2.37	0.55	0.44
TOS	Mean	0.21	0.15 *	0.24 ^†††^	0.17 ^‡^	0.22 ^††§^	0.16 *^‡‡‡#^	0.23 ^††§∧^
(μmol H_2_O_2_ Equiv./g)	SD	0.04	0.04	0.03	0.03	0.03	0.04	0.06
MDA	Mean	0.090	0.100	0.095	0.110	0.110	0.095	0.115
(μmol/g)	SD	0.040	0.140	0.080	0.030	0.020	0.050	0.070
SHG	Median	21.80	24.25	23.10	28.00 ***^‡^	24.60 ^#^	27.05 **^‡^	23.60 ^#∧^
(mmol/g)	Range	6.20–32.89	7.50–36.47	7.30–36.51	9.40–34.90	2.70–38.07	4.70–33.88	3.80–39.05
KIDNEY		Control	0.1	0.5	1	3	7	15
AOPP	Mean	6.75	6.04	4.66	6.86	5.56	7.40	5.71
(μmol/min/g)	SD	1.21	1.26	0.817	0.74	1.06	2.20	1.57
O_2_^−^	Mean	34.91	35.24	33.61	27.13 **^††‡^	33.64	29.13 **^††^	33.87 ^§∧^
(μmol/min/g)	SD	3.60	6.01	4.70	1.56	9.782	4.69	5.75
SOD	Mean	13.56	9.20 *	10.64	7.20 **	7.18 **	7.45 **	8.10 **
(U/g)	SD	0.95	2.36	3.44	2.80	4.24	2.53	3.23
TOS	Mean	3.30	3.30	3.60	3.11	3.84	3.35	3.78
(μmol H_2_O_2_ Equiv./g)	SD	0.38	0.37	0.34	0.20	0.41	0.71	0.66
MDA	Mean	1.66	1.42	1.02 **	0.73 ***	1.10 *	0.69 ***	1.14 *
(μmol/g)	SD	0.32	0.37	0.23	0.55	0.31	0.14	0.22
SHG	Median	0.08	0.09	0.07	0.09	0.09	0.11	0.09
(mmol/g)	Range	0.07–0.10	0.06–0.11	0.05–0.08	0.07–0.13	0.06–0.09	0.10–0.19	0.05–0.09

Advanced oxidation protein products (AOPP), super oxide anion (O_2_^−^), superoxide dismutase (SOD), total oxidative status (TOS), malondialdehyde (MDA), sulfhydryl groups (SHG); * ^† ‡ § # ∧^ *p* < 0.05; ** ^††^ *p* < 0.01; *** ^††† ‡‡‡^ *p* < 0.001; compared to control, 0.1, 0.5, 1, 3, and 7 group, respectively; ANOVA followed by Fisher’s LSD, or the Kruskal-Wallis test followed by the Mann–Whitney U test.

## Data Availability

Data will be available on the request.
